# Houston, we have a problem of opioid crisis… and Rome?

**DOI:** 10.1186/s44158-023-00121-7

**Published:** 2023-10-17

**Authors:** Sebastiano Mercadante

**Affiliations:** Main Regional Center for Pain Relief and Supportive/Palliative Care, La Maddalena Cancer Center, Via San Lorenzo 312, 90146 Palermo, Italy

**Keywords:** Opioids, Chronic pain, Perioperative pain

The abuse of opioid drugs in the USA represents a real emergency which has led to increased pressure on the health care system. In the previous 20 years, liberal opioid use was encouraged in an effort to improve patient satisfaction along with misguided efforts by the pharmaceutical industry acting as driving forces [1]. This resulted in a clear disconnect between the prescriptions provided to the patients and the opioids needed to manage pain [2]. Newspapers, books, magazines, and movies have largely emphasized the unbelievable sequence of events that have led to this endless crisis, although the prevalence of death is now principally due to street illicit drug trafficking. Despite reductions in opioid prescriptions (44% decrease since 2011), however, no reductions in drug-related mortality have been reported [3]. As pharmaceutical opioid prescribing rates declined following the 2016 guidelines, the Centers for Disease Control and Prevention (CDC) came under pressure for creating a barrier to care for patients who suffer from acute or chronic pain [4]; some significant changes in CDC 2016 recommendations have been released [5].

In the meantime in Italy, on the basis of the overseas opioid crisis, illegal drug trafficking, and some pressure from politicians, the National Regulatory Agency decided to label opioid packets with a red flag “opioids produce addiction,” despite the lack of alarming data. In a Mediterranean country like Italy, characterized by the lowest opioid consumption in Europe, news from the USA could potentially have catastrophic consequences, as such a decision could result in further underprescription of opioids and may amplify cultural barriers existing in Southern Europe, where we have to face exactly the opposite problem to that observed in USA [6]. For instance, Europe as a whole is not facing an opioid crisis and there are marked differences between European countries in trends of opioid prescribing and of proxies for opioid-related harms [7]. For comparison, while in the USA 19% of patients developed nonmedical opioid use behavior [8], in a study performed in Italy, no patient displayed clinical aberrant behaviors after a 1-month follow-up [9].

In patients with chronic pain, the risk of opioid-aberrant behavior has been often advocated for suggesting interventional procedures as the first line. Apart from the fact that it is common observation to find casual use of opioids even after such procedures, an analytic review on the use of opioids for the treatment of chronic non-cancer pain has shown scientific evidence of the effectiveness of opioids in treating pain and of high variability in opioid dose requirements and side effects. The prevalence of opioid use disorder associated with prescription opioids is likely < 3%. The estimated risk of death from opioid treatment involving doses above 100 MMED is ∼0.25%/year. A large number of studies refute the statement that short-term use of opioids to treat acute pain predisposes to the development of opioid use disorder. Due to a lack of comparative studies, there are no scientific grounds for considering alternative non-pharmacologic treatments as an adequate substitute for opioid therapy. Rather, these treatments might serve to improve opioid analgesic effects, thereby reducing dosage. Indeed, there are reasons to question the ostensible risks of co-prescription of opioids and benzodiazepines. It seems that the opioid crisis resides predominantly in the streets and that efforts to curtail it by constraining opioid treatment in the clinic are unlikely to succeed [10]. On the other hand, an appropriate prescription by pain specialists, observing the various guidelines released in these years, may prevent these risks [11].

Chronic analgesic therapy with opioids should be provided only by pain specialists to patients with clear medical necessity when providing dose stability with improvement in pain and function, alone or in combinations with other treatments, in low doses, and with appropriate adherence and adverse events monitoring. This approach has nothing to do with illicit or inappropriate prescriptions.

## Opioids in the perioperative period

Opioids are an important component of general anesthesia and perioperative analgesia. The opioid abuse epidemic has induced clinicians to move away from opioids toward other drugs for general anesthesia and postoperative pain treatment. Persistent opioid use after surgery is a component of the opioid epidemic and is a major concern for clinicians and public health officials. Data suggest that perioperative opioid usage patterns may contribute to opioid misuse after operation, although how the administration of intraoperative opioids may influence this problem is unclear [12, 13]. It has been reported that discharge opioid prescriptions may result in persistent opioid use and diversion. In the USA, about 1/4 of patients are receiving opioids before surgery and are more likely to be on opioids months after surgery [14], meaning that hundreds of thousands of patients are at risk for persistent opioid use after surgery*.* An increased enthusiasm to advocate opioid-free strategies has been developed in the last few years. A combination of known analgesics and adjuvants has been advocated for substituting opioids in the perioperative period. Existing data, however, indicate that opioid-free strategies do not fully acknowledge limits and gaps within evidence and clinical practice issues. In addition, this approach does not allow any dose titration based on patient needs and remains unclear about optimal components and their role in different surgical conditions and perioperative phases [15]. More importantly, it does not serve to decrease the risk of persistent opioid use [16], thereby distracting anesthesiologists from optimizing pain control and minimizing realistic long-term harms.

The major problems reside in the appropriate use of opioids in the perioperative period. Conditions such as those depicted in US studies are unlikely to be evident in Italy, where, in contrast, postoperative pain is often based on anti-inflammatory drugs. In my cancer center where potentially more patients receive opioids for their cancer pain, the finding of misuse is null, and in opioid-naive patients postoperative opioid prescription does not exceed 3 days, even for major cancer surgery, mostly performed under blended anesthesia. Regional anesthesia and minimally invasive surgery may reduce the need for postopertive analgesics, including opioids. Potential solutions to the problem of persistent opioid use after surgery should focus on proper ‘opioid stewardship’ after surgery, wherein opioids are used conservatively in combination with other analgesic adjuncts, and opioid prescribing for home use is limited [17].

## Data Availability

NA.
